# Improving the Properties of Porous Geopolymers Based on TPP Ash and Slag Waste by Adjusting Their Chemical Composition

**DOI:** 10.3390/ma15072587

**Published:** 2022-03-31

**Authors:** Elena A. Yatsenko, Boris M. Goltsman, Sergei V. Trofimov, Viktor M. Kurdashov, Yuri V. Novikov, Victoria A. Smoliy, Anna V. Ryabova, Lyudmila V. Klimova

**Affiliations:** The Department “General Chemistry and Technology Silicates”, Faculty of Technology, Platov South-Russian State Polytechnic University (NPI), Prosveshcheniya Street 132, Rostov Region, 346428 Novocherkassk, Russia; e_yatsenko@mail.ru (E.A.Y.); boriuspost@gmail.com (B.M.G.); viktorkurdashov@yandex.ru (V.M.K.); novikovtnv@yandex.ru (Y.V.N.); vikk-toria@yandex.ru (V.A.S.); annet20002006@yandex.ru (A.V.R.); lyudmila.clim@yandex.ru (L.V.K.)

**Keywords:** porous geopolymer, ash and slag waste, glass, sand, structure formation, Arctic zone

## Abstract

The possibility of improving the properties of porous geopolymer materials based on ash and slag waste from thermal power plants by adjusting their chemical composition is considered. An X-ray phase analysis of ash and slag wastes was carried out, the geopolymers’ precursor compositions were calculated, and additives to correct their chemical composition were selected. The samples were synthesized and their physical and mechanical properties (density, porosity, compressive strength, thermal conductivity) were analyzed. The micro- and macro-structure of the samples and the pore distribution of the obtained geopolymers were studied and pore-distribution histograms were obtained. The influence of Si:Al ratio on structural changes was described. The geopolymers’ phase composition was studied, consisting of an amorphous phase and high quartz and mullite. A conclusion about the applicability of this method for obtaining high-quality porous geopolymers was made.

## 1. Introduction

When coal is burned in thermal power plants, the non-combustible part of the coal forms waste (coal-combustion products), which is a mixture of fly ash, bottom ash and boiler slag. About 1 billion tons of ash and slag waste (ASW) are generated annually around the world, and therefore information about their recycling in different countries is great. In India, coal energy accounts for 72% of all electricity, while more than 215 million tons of ASW is generated annually, and their use is about 168 million tons. The largest volumes of waste are used in the production of cement (28%), land reclamation (13%), construction of dams (10%), production of bricks and tiles (10%), etc. [1].

In the USA, more than 70 million tons of ASW are produced annually, and the volume recycled is 60%. The main areas of their processing are the production of cements and concretes (40%), the construction of dams (10%), etc. [2]. In China, about 200 million tons of ASW are produced annually. The volume of waste recycled in this country is 65%, while the largest share of ash and slag is used in the cement industry (25%), in obtaining budget building materials (18%), and in use as an additive for concrete (10%) [3].

In EU countries, 40 million tons of ASW are produced annually, while the volume of them recycled is almost 90%. The main part of ash and slag is used as an additive to concrete (41%), in cement production (34%), and in road construction (16%) [4].

In the Russian Federation, about 22 million tons of combustion waste is generated annually at coal-generation facilities [5,6]. According to various estimates, 1.4–1.8 billion tons of ASW are currently accumulated at ash dumps in the Russian Federation, occupying vast areas of more than 20,000 km^2^ [7,8].

Ash and slag dumps are often designed near the territory of a thermal power plant and near a residential area, which causes significant harm to the environment, and therefore it is relevant to search for new ways of recycling and processing ash and slag into promising materials for various purposes.

In the Russian Federation, the volume of ASW processing reaches 10–12% [9,10], and they are mainly used for the needs of the cement industry, where they are used as an additive for Portland cement [11,12,13,14]. At the same time, it is known that the volume of ash and slag being processed in Germany reaches 100% [15], in India—more than 60% [16], in the countries of the European Union—about 90% [17], in the USA—25% [18].

According to the chemical and phase-mineralogical composition, ASW is a complex mixture which contains 45–60% SiO_2_, 10–30% Al_2_O_3_, as well as compounds of calcium, magnesium, iron, sulfur, etc. [19]. In view of the high content of aluminosilicate amorphous phase in the composition of ash and slag and their high dispersion, the method of processing ash and slag into geopolymers, which are a new class of materials that are hydraulic binders of alkaline activation, is promising [20,21,22]. They are glass–crystalline aluminosilicates consisting of [SiO_4_] and [AlO_4_] tetrahedra connected in series by bridging oxygen. Geopolymer materials have found application in construction, but this is very limited, mainly due to the impossibility of predicting technological parameters of production and performance properties of geopolymers due to the use of raw materials of variable composition [23,24,25,26]. The empirical formula of a geopolymer can be represented as follows: M_n_{(SiO_2_)_z_AlO_2_}_n_,wH_2_O, where M is an alkali metal atom, z is the Si/Al ratio equal to 1, 2, or 3, and n is the degree of polymerization or polycondensation [27]. The numbers denoted by the letter “z” distinguish three types of geopolymer: poly(sialate), with a Si/Al ratio of 1:1, poly(sialate-siloxo) with a Si/Al ratio of 1:2, and poly(sialate-disiloxo), with a Si/Al ratio of 1:3 [28].

The problem of accumulation of ASW is especially acute in the Arctic zone of the Russian Federation, since the volume of coal generation in this territory is more than 20% [29]. In this regard, the issue of ash and slag recycling in this region is relevant. Since geopolymer materials are highly frost-resistant and can withstand up to 150 thaw–freeze cycles [30,31,32], their use in road construction under permafrost and extreme climatic conditions is promising [22].

It is known [33,34] that the main problem associated with road construction in the Arctic zone is swelling of the soil due to the freezing of the moisture contained in it. This is due to waterlogging of the soil and long periods with a negative ambient temperature. This factor leads to an uneven rise of the soil and the destruction of the roadway, and therefore an additional frost-protective layer of pavement is required. In the future, geopolymer materials obtained from local technogenic raw materials can be used as a frost-protection layer [35]. Such a solution can solve the problem of ASW recycling by processing them into a useful product.

Research in the field of production of geopolymer materials based on ASW is quite diverse. As indicated in [36], 48% of researchers used fly ash as the main raw material, and 23% used slag. Most often, dense concrete-like materials are obtained on the basis of geopolymer compositions [37,38]. These geopolymer binders can be used to obtain a whole range of products, including pipelines [39]. A significant difference in the present study is the development of a method for adjusting the chemical composition of the geopolymer by introducing silica-containing additives, which change the ratio of Si:Al and Si:Na. In the course of earlier work, the possibility of using ASW from the Apatitskaya combined heat and power plant (CHPP) and Severodvinskaya CHPP-1, located in the Arctic zone of the Russian Federation, as a raw material, was determined. These power plants were selected as having the largest power capacity in the study region, and therefore, producing the largest amount of waste. A number of physical–chemical studies on the considered ash and slag were carried out. Chemical analysis, differential thermal analysis, and X-ray phase analysis were carried out, and the microstructure of the samples was determined. It was determined that due to the presence of an amorphous aluminosilicate structure in the composition of ash and slag, they can be used in the synthesis of geopolymer materials [40]. Radiological studies of ASW were carried out, during which it was found that the samples comply with regulatory requirements and can be used for all types of construction. The synthesis of pilot compositions of geopolymers was carried out, and the optimal amounts of an alkaline activator and foaming additives were determined. It was revealed that samples based on the ASW of the Apatitskaya CHPP do not have the required operational qualities and cannot withstand even a slight mechanical impact. According to the authors, this factor is associated with insufficient content of SiO_2_ in the waste composition. In this regard, the purpose of this work is to study the effect of additives that increase the content of SiO_2_, such as glass cullet and quartz sand, on the physical and mechanical properties of the obtained geopolymers.

## 2. Materials and Methods

### 2.1. Materials

**Figure 1:**
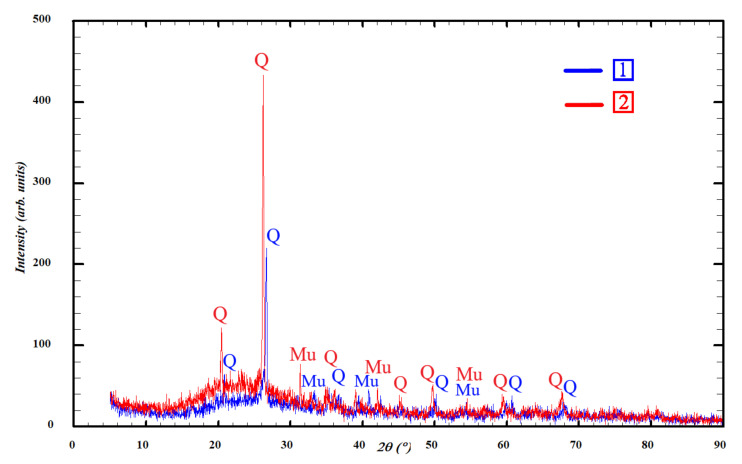
Results of X-ray analysis of ASW: 1—Apatitskaya CHPP; 2—Severodvinskaya CHPP-1; Q—high quartz, Mu—mullite.

To study the effect of the chemical composition of ASW on the physical–chemical and mechanical properties of porous geopolymers, ASW from the Apatitskaya CHPP (Apatity, Murmansk Region, Russia) and Severodvinskaya CHPP-1 (Severodvinsk, Arkhangelsk region, Russia) were studied. Previously, the authors studied the chemical (Table 1) and phase (Figure 1) composition of the used ASW [40]. As can be seen, the main components in both cases are SiO_2_ and Al_2_O_3_, the total content of which is more than 75%. The SiO_2_/Al_2_O_3_ ratio at the Apatitskaya CHPP is 2.37 and of SiO_2_/Na_2_O = 66.91; at the Severodvinskaya CHPP-1 these are 3.44 and 17.15, respectively. The content of CaO in the studied ASW is less than 10%, which defines them as being low-calcium. The crystalline phases represented are silicon dioxide (SiO_2_) in the form of high quartz (ICCD PDF# 82-0512) and mullite (ICCD PDF# 15-0776). The true density of ASW from the Apatitskaya CHPP is 1831 kg/m^3^, and from the Severodvinskaya CHPP-1 it is 2034 kg/m^3^.

It is known that the Si/Al and Si/Na ratios are the determining factors in the formation of geopolymer materials’ properties [41,42]. Thus, it was planned to increase this ratio by increasing the content of SiO_2_ and Na_2_O in aluminosilicate raw materials, which was achieved by the preparation of a precursor mixture with the addition of quartz sand from the Karpov-Yarskoye deposit (Millerovo, Rostov region, Russia) and cullet of white container glass, the composition of which is also shown in Table 1. All the listed raw materials were dried to constant weight, after which they were crushed to a particle size of less than 250 µm.

For alkaline activation of aluminosilicate components, a mixture of waterglass (sodium hydrosilicate, silicate modulus = 2, water content 55 wt.%, Sil-Ex, Asbest, Russia) and NaOH solution (LenReactive, St. Petersburg, Russia) was used as an activating agent. To prepare the solution, a separate container was used in which a pre-weighed sample of NaOH powder with a purity of 99% and deionized water were mixed to obtain a molar concentration of 12 mol/l (12M).

### 2.2. Calculation of Compositions and Synthesis of Porous Geopolymers

To calculate the theoretical ratio of “ASW / modifying component (glass or sand)”, the method of calculating a batch mixture with a given chemical composition was used. As mentioned above, components with chemical compositions presented in Table 1 were used as raw materials. It was decided to increase the content of SiO_2_ of the Apatitskaya ASW to the values of the Severodvinskaya ASW.

According to the calculations, to increase the content of the above oxide in the chemical composition, the following ratios were obtained of the main raw material (reduced to 100%): “Apatitskaya ASW:glass” = 70:30 and “Apatitskaya ASW:sand” = 80:20.

To calculate the chemical compositions of the synthesized porous geopolymers, the presented component composition of the raw mixture (Table 2) was used with recalculation of all components per 100% of precursor. In “S” Composition, the geopolymer was obtained on the basis of ASW from the Severodvinskaya CHPP-1, and in “A” Composition, on the basis of ASW from the Apatitskaya CHPP. The “s” symbol in the “As” Composition denotes the addition of sand, and the “g” symbol in the “Ag” Composition indicates the addition of glass. Based on the above, calculation results of the chemical compositions of porous geopolymers were obtained, recalculated to 100%, and presented in Table 3.

The prepared 12M NaOH solution was mixed with a sodium waterglass. Then, the resulting suspension was poured into an ASW powder. Stirring of the geopolymer suspension was carried out for 600 s in a laboratory mill (MSL-1S, PromStroyMash, Moscow, Russia) at 120 rpm in ceramic drum with the ratio “geopolymer precursor: grinding bodies” = 1:1.5. After the preparation of the mixture, a foaming agent (aluminum powder, «SouthReagent», Rostov-on-don, Russia) was added to the compositions, after which the mixture was stirred for another 30 s under similar conditions [43]. Next, the resulting geopolymer suspension was poured into cubic molds with an edge length of 30 mm and sent for curing. The curing of the geopolymer precursor was carried out at room temperature, 24 ± 2 °C, and relative air humidity of 62 ± 5% for 2 h. After that, the samples were placed in a drying oven (SS-80-01 SPU, Smolenskoye SKTB SPU, Smolensk, Russia) and heated to a temperature of 60 ± 2 °C for 24 h. Then, samples were demolded and cured for 72 h at room temperature, 25 ± 2 °C, and relative air humidity of 58 ± 5% [44,45].

### 2.3. Methods

The linear dimensions of the samples after curing were determined with a caliper with an accuracy of ±0.1 mm, after which the volume V of the sample was calculated from their values by multiplying the length of the geopolymer by its width and height. The mass of the samples was measured with an accuracy of 0.01 g. The density of the samples d, kg/m^3^, was determined as the ratio of the mass to the volume of the sample according to Equation (1):

d = m/V·1000, kg/m^3^ (1)

where m—sample mass, g; V—sample volume, cm^3^.

Porosity P, %, shows the volume of pores in a porous material, which is defined as the ratio of bulk density d_b_ to the true density d_t_ in the synthesized geopolymer. Porosity was calculated according to Equation (2):

P = (1 − d_b_/d_t_)·100, % (2)

where d_b_—sample bulk density, kg/m^3^; d_t_—sample true density, kg/m^3^.

Foam expansion E_f_,% was calculated according to Equation (3) [46]:

E_f_ = (h_2_ − h_1_)/h_1_·100, % (3)

where h_1_—geopolymer precursor height after molding, cm; h_2_—resulting geopolymer height after curing, cm.

The strength characteristics of the samples were determined using a test press (TP-1-350, TestPress, Misailovo village, Russia) with a force measurement range of 0.1 to 350 kN with a measurement accuracy of ±2% in the range of 0.1 to 7 kN, and ±1%—from 7 to 350 kN. The compressive strength of the samples R, MPa, was determined by Equation (4):

R = 1000·P/S, MPa(4)

where P—breaking load, kN; S—sample area, cm^2^.

Thermal conductivity was studied using an ITP-MG4.03/5(III) POTOK heat flow and temperature meter (OOO SKB Stroypribor, Chelyabinsk, Russia) at an ambient temperature of 23 ± 2 °C and relative air humidity of 55 ± 5%. The allowable limit of the relative error in measuring the heat-flow density is ±6%, and for temperature it is ±0.2 °C in the range from −30 to +80 °C. Heat-flow sensors consist of series of connected galvanic copper–constanton thermocouples. The temperature sensors are platinum resistance temperature sensors enclosed in a sealed metal case. The sample surface was cleaned to eliminate visible and palpable roughness, after which thermal paste Z3 (>1.134 W/m K, Deepcool, Beijing, China) was applied to exclude an air gap in the measurement area. Plasticine was used to attach the sensors.

Each recorded test value is the average of 3 measurements.

Pore size and distribution were determined by high-resolution microphotography using Nano Measurer 1.2. software.

The EDS spectra of the chemical composition were studied on a FEI Quanta 200 scanning electron–optical microscope (FEI Company, Hillsboro, OR, USA) with an EDAX Element Energy-Dispersive Spectroscopy (EDS) System (AMETEK, Berwyn, PA, USA) microanalysis system in the following range: voltage 20 kV, magnification (MAG) 1000×, amp-time (µs): 3.84.

The phase composition of the synthesized samples was determined using powder X-ray phase analysis (XRD). The samples were crushed and examined using an ARLX’TRA X-ray diffractometer (Thermo Fisher Scientific, Waltham, MA, USA). The characteristic radiation of a copper anode was used (wavelengths CuKα1 1.5406 Å, CuKα2 1.5444 Å). Shooting conditions: 35 kV, 30 mA. Data interpretation was carried out using the Crystallographica Search-Match Version 3 software package of the ICDD PDF 2 database (International Center for Diffraction Data).

Microstructure analysis was performed using a JEOL JSL 5300 scanning electron microscope (JEOL, Tokyo, Japan) operating at 5 keV, configured to use secondary electron-backscattering detectors. This equipment is a part of the “Nanotechnologies” CCU of the Platov South-Russian State Polytechnic University (NPI).

## 3. Results and Discussion

### 3.1. Macrostructure and Properties

**Figure 2:**
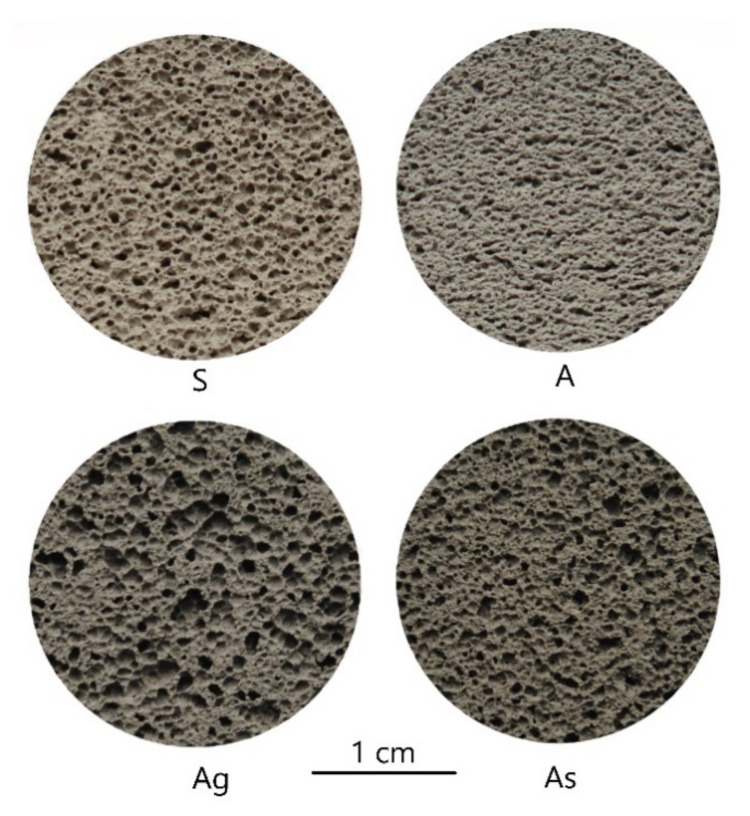
Macrostructure of synthesized geopolymers.

**Figure 3:**
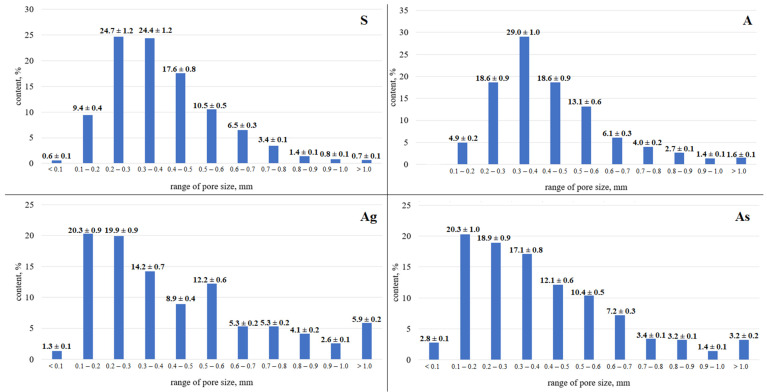
Histogram of Pore Size Distribution in Geopolymer Samples.

On the basis of the presented component mixtures and the developed technology described earlier, samples with a structure were obtained. The internal macrostructure of the synthesized samples is shown in Figure 2. According to Equations (1)–(4), the main characteristics of the synthesized porous geopolymers were calculated, as presented in Table 4. Figure 3 shows histograms of the distribution of pore sizes in the studied samples.

The sample of the “S” series has the highest strength compared to the samples of other series, which is mainly due to the chemical composition of the ASW used. The sample has the highest total porosity and uniform distribution of macropores. Sample “S” is dominated by macropores with a diameter of 0.2–0.4 mm.

The sample of the “A” series is characterized by low strength, which is lower than the strength of the “S” series sample by more than two times. The bulk density of the sample is the highest among the studied compositions, which is associated with a low expansion coefficient. The distribution of macropores is uniform. The sample is dominated by pores with a diameter of 0.3–0.4 mm.

The “Ag” series sample has an average strength two times higher than the “A” series samples. This factor clearly reflects the positive effect of glass on the properties of the geopolymer. The distribution of macropores in the sample is uniform. In the “Ag” sample, pores with a diameter of 0.1–0.3 mm predominate, and numerous macropores with a diameter of 0.5–0.6 mm are also observed.

The sample of the “As” series has a higher strength compared to the sample of the “A” series, but less than the samples of the “Ag” series, which indicates a positive effect of sand on the physical and mechanical properties of the geopolymer material. However, as can be seen, glass is the most preferred material to achieve the goal, since it has an amorphous structure similar to the structure of amorphous aluminosilicate phases in ASW. The average strength of a sample of the “As” series is the lowest among the studied compositions. The distribution of macropores is uniform. The “As” sample is dominated by pores up to 0.1–0.4 mm in diameter.

**Figure 4:**
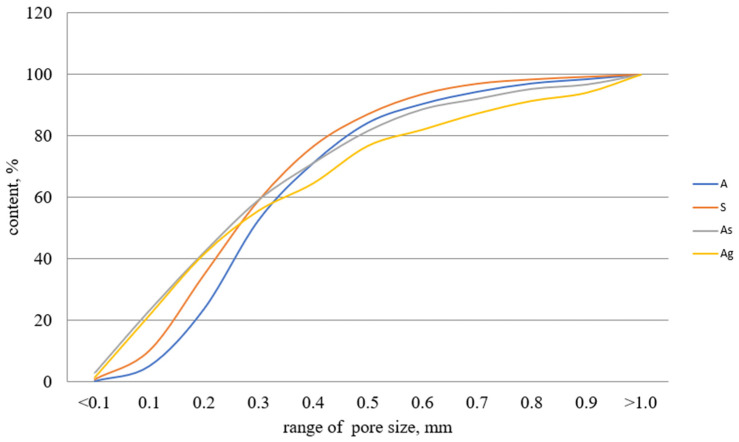
Comparison of pore size distribution in geopolymer samples.

Figure 4 shows a graph comparing the distribution of pore sizes in all samples.

This graph was obtained as a result of a comparative analysis of the distribution of pore sizes in the studied samples, and confirms the above conclusions that the samples predominantly contain pores up to 0.4 mm. It can be seen that the proportion of pores of this size is 70.5% on average. The graph also confirms the data obtained within macroscopic analysis of the pore size. In particular, the “Ag” sample that contains some large pores shows a smoother growth on the graph in the range of over 0.4 mm, which indicates an increase in the number of pores of a given size.

**Figure 5:**
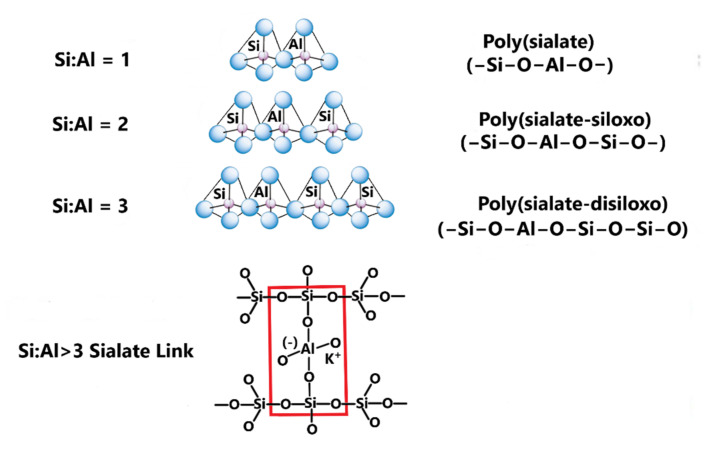
Possible geopolymer structures.

Geopolymers have a structure with silicon and aluminum atoms repeating in chains. Depending on the alternation of silicon and aluminum atoms, geopolymers are divided into poly(sialate), poly(sialate-siloxo), or poly(sialate-disiloxo) (Figure 5). Structural elements of sialates are in the form of tetrahedra, in the center of which are silicon and aluminum atoms bonded to four oxygen atoms. These elements are able to form two- and three-dimensional structures. During synthesis, silicon and aluminum atoms form strong, branched Si–O–Al–O chains, due to which geopolymers are not inferior in physical and mechanical properties to rocks [21].

According to calculations, the Si:Al ratio in the synthesized compositions decreases in the following order: S (Si:Al = 2.94) → Ag (Si:Al = 2.76) → As (Si:Al = 2.73) → A (Si:Al = 1.95). The structure of geopolymers shifts from poly(sialate-disiloxo) to poly(sialate-siloxo). Such displacement further reduces the strength of geopolymers, since it is known that an increase in the amount of Al makes it difficult to form a stable structure. However, it should be noted that the parameters of the porous structure (size and distribution of pores) still have the main influence on the strength.

### 3.2. Phase Composition

**Figure 6:**
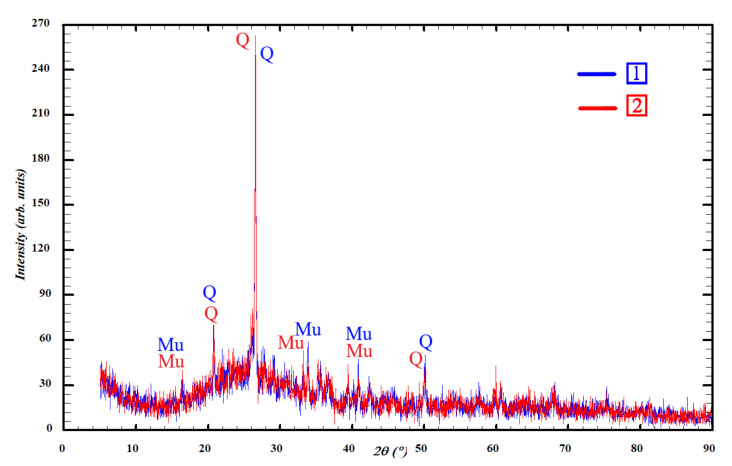
X-ray diffraction pattern of porous geopolymers based on the Apatitskaya CHPP: 1—“Ag” Composition, 2—“As” Composition; Q—high quartz, Mu—mullite.

The X-ray diffraction analysis presented in Figure 6 was carried out in order to study the crystalline peaks necessary to identify the phases associated with the formation of porous geopolymers.

It can be seen from the obtained results that the X-ray diffraction patterns of both compositions are very similar and strongly superimposed on each other. Analyzing the presented X-ray patterns, we can conclude that both studied modifying additives are characterized by low peak intensity due to the low content of crystalline phases, represented by silicon dioxide (ICCD PDF# 82-0512) and mullite (ICCD PDF# 15-0776). The identified crystalline phases are the main components of the formation of porous geopolymers. Additionally, there is a significant amount of X-ray amorphous glass phase in both compositions presented in ASW and glass. Its presence is confirmed by an amorphous “halo” in the 2θ shooting angles ranging from 14 to 38°, which indicates the presence of an amorphous structure in the form of an aluminosilicate glass phase. The use of glass instead of sand as a modifying additive reduced the intensity of the quartz and mullite peaks by an average of 10%. This is due to the excess internal energy of the glass, which causes its high reactivity and intensity of interaction with the alkaline activator. Thus, the resulting geopolymers in terms of phase composition are glass–ceramic materials. Such a structure provides increased strength in comparison with glass materials.

### 3.3. Microstructure

**Figure 7:**
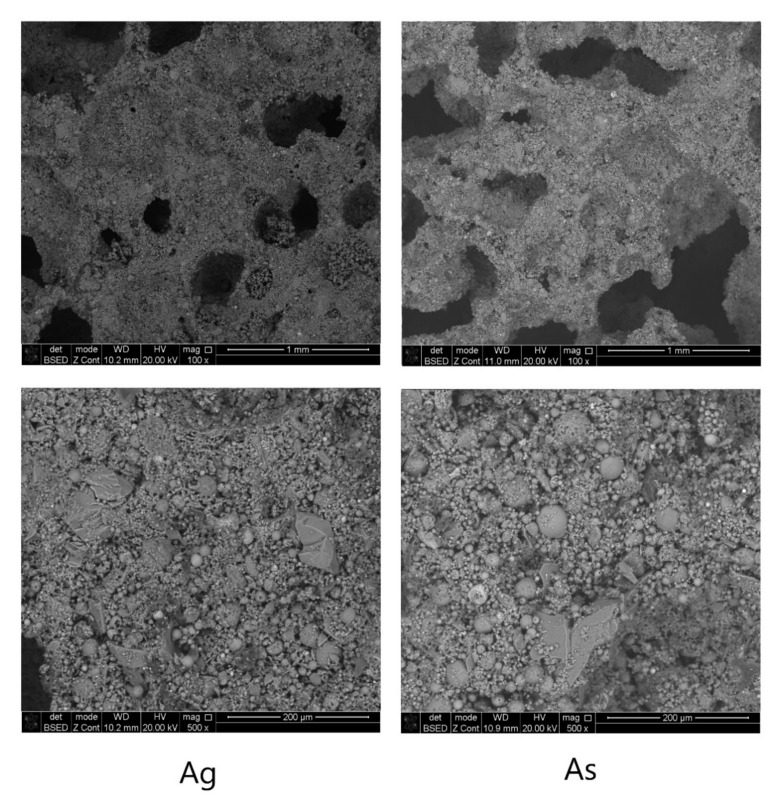
Microstructure of geopolymers.

The microstructures of the synthesized geopolymers based on the ASW of the Apatitskaya CHPP with the addition of glass and sand are shown in Figure 7.

**Figure 8:**
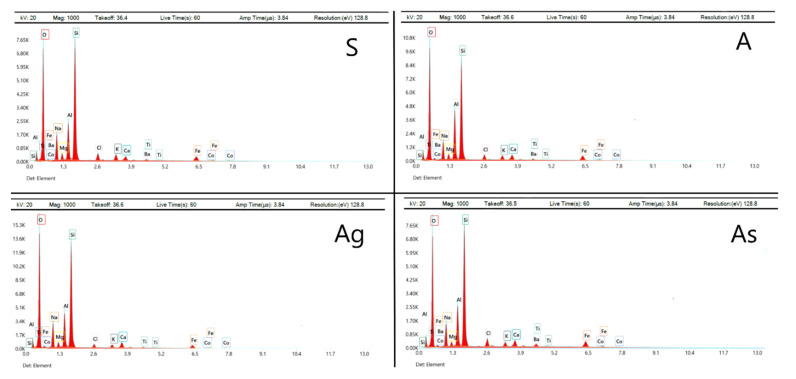
EDS spectra of synthesized geopolymers.

The “As” Composition with the addition of glass powder is characterized by high pore formation and uneven distribution of macropores. The sizes of the observed pores are in the range of 0.2 to 0.5 mm. The “As” Composition with the addition of sand is also characterized by high pore formation and a more uneven distribution of macropores. The sizes of the observed pores are in the range from 0.3 to 1 mm. An analysis of the structure of the pore walls made it possible to discover that the material of the walls is mainly represented by particles of a spherical shape—hollow aluminosilicate glass–crystalline ash microspheres formed during high-temperature combustion at thermal power plants. Additionally, particles of an acute-angled irregular shape were found—particles of the slag (bottom ash) component of the ASW mixture, as well as crystalline sand particles and ground glass particles. Thus, a geopolymer is a polycrystalline material consisting of spherical and acute-angled particles bonded together by the reaction products of an alkali activator and aluminosilicate main raw materials. The results of EEDS analysis are represented in Table 5 and Figure 8.

As can be seen from the data obtained, geopolymers mainly consist of O, Si, Na, and Al. These results confirm the results of theoretical calculations of geopolymer chemical compositions.

## 4. Conclusions

The conducted studies have shown that glass cullet and quartz sand additives have a positive effect on the properties of porous geopolymer materials. The best additive that affects the strength of the geopolymer material based on the ASW from the Apatitskaya CHPP is glass, which is obviously associated with the amorphous structure of the glass similar in properties to the aluminosilicate amorphous phases in the ASW. It was found that the strength of the obtained geopolymer with the addition of glass increased by more than two times in comparison with a sample without additives. The strength of the sample containing sand increased by 1.8 times. The porous structures of all the studied samples are uniform and mostly represented by macropores up to 0.5 mm in size, which is displayed on the histograms of the pore size-distribution ranges. Microscopic analysis showed that the geopolymer is a polycrystalline material consisting of spherical and acute-angled particles bonded by the reaction products of an alkaline activator and aluminosilicate raw materials. On the presented XRD curves of the synthesized geopolymer materials, the presence of a glassy phase, represented by a “halo”, is observed, as well as peaks indicating the presence of silicon dioxide in the form of high quartz, and mullite.

Analyzing the above, we can conclude that it is possible to correct the precursor compositions of geopolymer materials used for road construction in the Arctic zone of the Russian Federation in order to improve their physical and mechanical properties.

## Figures and Tables

**Table 1:** Chemical composition of raw materials, wt.%.

Component	SiO_2_	Al_2_O_3_	Fe_2_O_3_	MgO	Na_2_O	K_2_O	CaO	TiO_2_	MnO	P_2_O_5_	SO_3_	LOI
ASW (Apatitskaya CHPP)	52.86	22.35	7.8	2.65	0.79	1.96	3.62	1.06	0.07	0.36	0.37	6.11
ASW (Severodvinskaya CHPP-1)	61.57	17.91	6.01	2.75	3.59	2.32	2.1	0.83	0.07	0.21	0.32	2.32
Glass	71.2	2.7	0.8	7.6	13.2	0.8	3.4	–	–	–	0.2	0.1
Quartz sand	98.91	0.29	0.07	–	–	–	–	–	–	–	–	0.73
Waterglass	29.2	0.61	0.1	–	14.26	–	0.2	–	–	–	0.11	55.52
NaOH	–	–	–	–	77.5	–	–	–	–	–	–	22.5

**Table 2:** Component composition of the geopolymer precursor, wt.%.

#	ASW	Addition	NaOH (Powder)	Water	Waterglass	Aluminum Powder, over 100
S	70.0(S)	-	2.5	5.0	22.5	2.0
A	70.0(A)	-	2.5	5.0	22.5	2.0
Ag	49.0(A)	21.0 (glass)	2.5	5.0	22.5	2.0
As	56.0(A)	14.0 (sand)	2.5	5.0	22.5	2.0

**Table 3:** Chemical compositions of initial and modified porous geopolymer precursors, wt.%.

#	SiO_2_	Al_2_O_3_	Fe_2_O_3_	MgO	Na_2_O	K_2_O	CaO	TiO_2_	MnO	P_2_O_5_	SO_3_	LOI
S	49.67	12.67	4.23	1.93	7.66	1.62	1.52	0.58	0.05	0,15	0.25	19.67
A	43.57	15.78	5.48	1.86	5.70	1.37	2.58	0.74	0.05	0.25	0.28	22.34
Ag	47.42	11.66	4.01	2.89	8.31	1.13	2.53	0.52	0.03	0.18	0.25	21.07
As	49.91	12.75	4.42	1.49	5.59	1.10	2.08	0.60	0.04	0.20	0.23	21.59

**Table 4:** Characteristics of the synthesized samples.

#	Foam Expansion,%	Density, kg/m^3^	Compressive Strength, MPa	Porosity, %	Thermal Conductivity, W/(m·K)
S	89.00 ± 2.96	510 ± 18	1.39 ± 0.05	74.93 ± 2.24	0.1057 ± 0.0004
A	74.88 ± 2.08	568 ± 23	0.61 ± 0.03	68.99 ± 2.84	0.1247 ± 0.0005
Ag	85.83 ± 1.06	516 ± 3	1.22 ± 0.06	71.83 ± 0.14	0.1408 ± 0.0002
As	77.37 ± 3.86	484 ± 12	1.10 ± 0.03	73.57 ± 2.27	0.1439 ± 0.0004

**Table 5:** Elemental composition of samples, wt.%.

Element	S	A	Ag	As
O	49.31	51.61	52.73	49.89
Na	9.16	7.05	9.55	8.16
K	1.92	1.50	1.08	1.32
Ca	1.53	1.97	2.22	2.26
Mg	1.59	1.46	1.48	1.07
Al	7.51	10.20	7.62	7.76
Si	22.06	19.85	21.00	21.22
Fe	3.66	3.67	2.48	4.41
Ba	0.75	0.75	–	0.77
Co	0.32	0.32	0.33	0.33
Ti	0.26	0.26	0.53	0.81
Cl	1.93	1.36	0.98	2.00

## Data Availability

All data presented in this study are included in the published article.
